# Alterations in detrusor contractility in rat model of bladder cancer

**DOI:** 10.1038/s41598-020-76653-7

**Published:** 2020-11-12

**Authors:** Igor B. Philyppov, Ganna V. Sotkis, Aurelien Rock, Morad Roudbaraki, Jean-Louis Bonnal, Brigitte Mauroy, Natalia Prevarskaya, Yaroslav M. Shuba

**Affiliations:** 1 Bogomoletz Institute of Physiology of the National Academy of Sciences of Ukraine, Bogomoletz Str., 4, Kyiv, 01024 Ukraine; 2 Laboratory of Cell Physiology, Inserm U1003, University of Lille, Villeneuve d’Ascq, France

**Keywords:** Biophysics, Cancer, Oncology, Urology

## Abstract

Urinary incontinence of idiopathic nature is a common complication of bladder cancer, yet, the mechanisms underlying changes in bladder contractility associated with cancer are not known. Here by using tensiometry on detrusor smooth muscle (DSM) strips from normal rats and rats with bladder cancer induced by known urothelial carcinogen, N-butyl-N-(4-hydroxybutyl)nitrosamine (BBN), we show that bladder cancer is associated with considerable changes in DSM contractility. These changes include: (1) decrease in the amplitude and frequency of spontaneous contractions, consistent with the decline of luminal pressures during filling, and detrusor underactivity; (2) diminution of parasympathetic DSM stimulation mainly at the expense of m-cholinergic excitatory transmission, suggestive of difficulty in bladder emptying and weakening of urine stream; (3) strengthening of TRPV1-dependent afferent limb of micturition reflex and TRPV1-mediated local contractility, promoting urge incontinence; (4) attenuation of stretch-dependent, TRPV4-mediated spontaneous contractility leading to overflow incontinence. These changes are consistent with the symptomatic of bladder dysfunction in bladder cancer patients. Considering that BBN-induced urothelial lesions in rodents largely resemble human urothelial lesions at least in their morphology, our studies establish for the first time underlying reasons for bladder dysfunction in bladder cancer.

## Introduction

Bladder cancer is the most common urinary tract malignancy. More than 90% of bladder tumors are urothelial carcinoma (the rest are represented by squamous cell carcinoma and adenocarcinoma), with approximately 420,000 new cases and 160,000 deaths registering worldwide annually, and about threefold higher incidence in men than women^1,2^. Exposure to environmental carcinogens and especially tobacco smoking represent the major risk factors. Although the most common bladder cancer symptom is haematuria, the patients also experience altered urination patterns (urgency, frequency and dysuria)^3,4^ indicative of the development of urinary incontinence. Such symptoms of urinary incontinence as frequency of urination above usual, feeling of urgency even when the bladder is not full, trouble urinating and weak urine stream are early signs of bladder cancer^4^. Generally, these signs and symptoms may also result from a number of non-cancerous pathologic condition, however, underlying reasons and mechanisms specifically related to bladder cancer have not been explored.

The factors contributing to urinary incontinence and loss of bladder control may be neurogenic (sensory or motor), myogenic, mixed, or idiopathic in origin^5–7^ with the term “idiopathic” reflecting the present lack of knowledge concerning the exact mechanisms of urinary bladder dysfunction (UBD) and is usually applied to a wide spectrum of different conditions that may have a common final pathophysiologic outcome^8^. Bladder cancer can definitely be viewed as one of such conditions.

Among ion channels participating in regulation of bladder detrusor smooth muscle (DSM) contractility the members of Ca^2+^-dependent potassium (K_Ca_)^9^ and transient receptor potential (TRP) families^10,11^ have emerged as particularly important. In the present study by using rat model of bladder cancer induced by known urothelial carcinogen, N-butyl-N-(4-hydroxybutyl)nitrosamine (BBN), which is a downstream metabolite of N-nitrosodibutylamine, found in tobacco smoke^12^, we asked how bladder cancer affects DSM contractility dependent on the activation of heat-sensitive TRPs of vanilloid subfamily, TRPV1, TRPV2 and TRPV4, and large conductance (BK) member of K_Ca_ family.

## Results

### Rat model of bladder cancer

**Figure 1:**
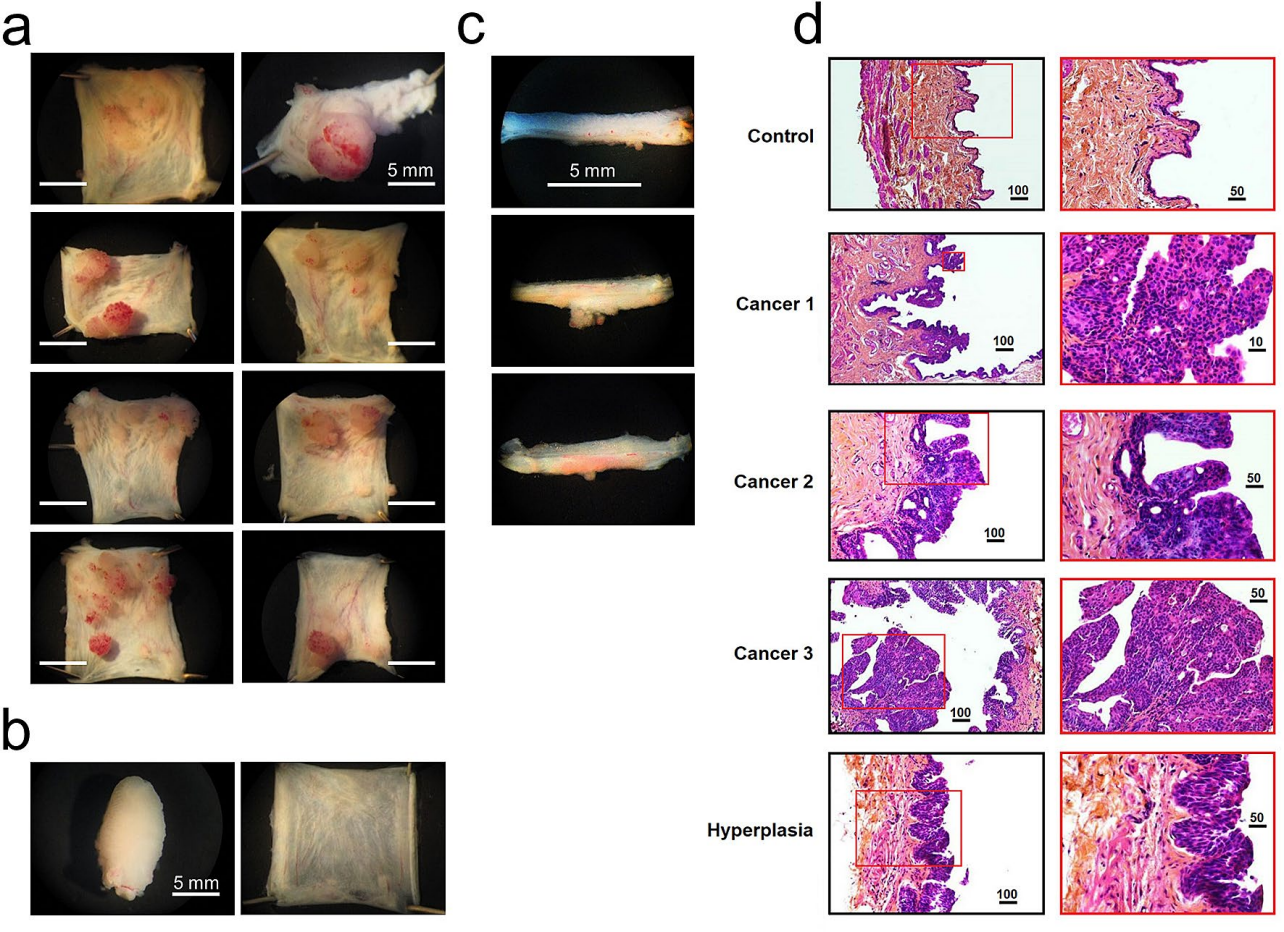
Macroscopic appearance of the rat bladder in rat model of BBN-induced bladder cancer. (**a**) Representative images of incised and everted bladders of the rats exposed to BBN in drinking water for 16 weeks, showing the heterogeneity of bladder cancer lesions. (**b**) For comparison normal intact (left) and incised (right) rat bladder is presented. (**c**) Examples of detrusor smooth muscle (DSM) strips obtained from the bladders of rats with BBN-induced bladder cancer used for contraction measurements; note papillary-type outgrowths. (**d**) Histopathological evaluation of lesions identified by H&E stain in rat BBN-induced bladder cancer, from top to bottom: normal urothelium (control), papillary carcinomas showing various degree of dysplasia (cancer 1–3) and simple hyperplasia; right images represent magnified views of boxed parts on the left; calibration bars are in micrometers.

Bladder cancers are classified onto 2 major subtypes based on how they grow: papillary carcinoma and flat urothelial carcinoma in situ (CIS) devoid of papillary structures^13^. High-grade invasive tumours growing into deeper layers of the bladder are usually defined as urothelial carcinoma with mixed histologic features (divergent differentiation)^13^. Papillary tumours exist in multiple morphologic variants, but only the so called micropapillary one is attributed to the infiltrating urothelial carcinoma, whereas all others are categorized as superficial tumour which usually does not invade or metastasize^13^. In our hands, the BBN model of rat bladder cancer after 4 months of BBN treatment mostly evolved into highly vascularized papillary-type lesions of variable dimensions, ranging from barely visible by naked eye submillimetre sizes to the ones occupying the entire bladder (Fig. 1a). In sharp contrast, normal (i.e. control) rat bladders displayed uniform intravesicular lining with only few bigger vessels discernible by naked eye (Fig. 1b).

Strips prepared from cancer-affected bladders commonly had visible “finger-like” growths (Fig. 1c). If the growths were too big they were cut off close to the base to enable strip positioning in the tensiometric arrangement.

Histopathological evaluation of bladder tissue samples from BBN-treated rats revealed multiple forms of microscopic lesions: simple hyperplasia, papillary and nodular hyperplasia, papilloma, and non-invasive papillary tumour of low malignant potential (Fig. 1d).

### Spontaneous activity

**Figure 2:**
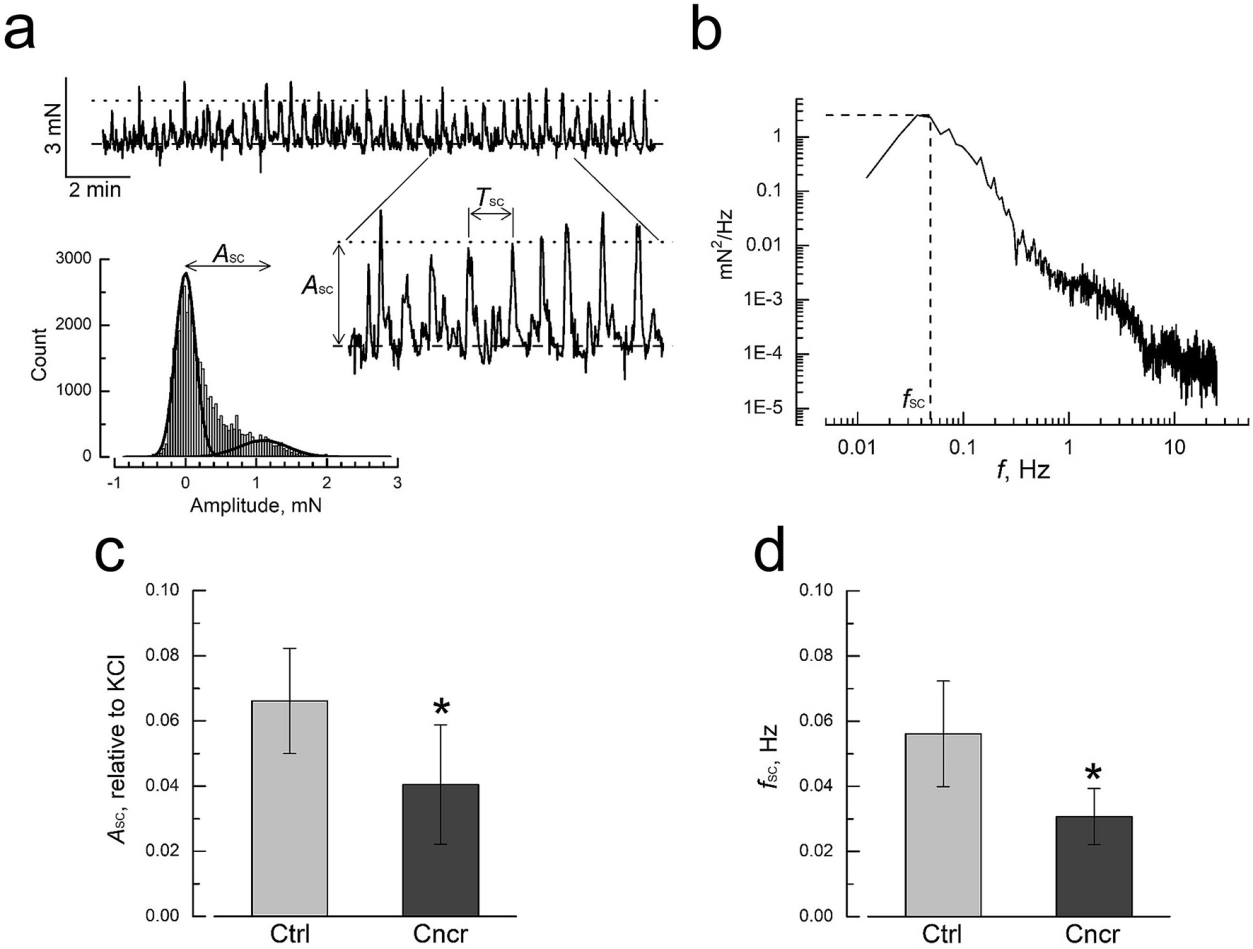
Bladder cancer reduces spontaneous contractions. (**a**) Representative recording of spontaneous contractions of DSM strip from control rat (top) with the part of the recoding presented at expanded amplitude and time scales (bottom right); *A*_SC_ and *f*_SC_ designate amplitude and frequency of spontaneous contractions, respectively, with *f*_SC_ = 1/*T*_SC_, where *T*_SC_ is the period of spontaneous contractions; dashed and dotted lines correspond to the baseline and *A*_SC_ levels, respectively; bottom left panel presents amplitude histogram of the recording with two Gaussians fit to the histogram; the distance between Gaussians maximums corresponds to *A*_SC_. (**b**) Typical power spectrum of spontaneous contractions used to estimate *f*_SC_. (**c**,**d**) Bar graphs showing the decrease of the amplitude (**c**) and frequency (**d**) of spontaneous contractions in cancer (Cncr, dark gray, n = 9) vs. normal (Ctrl, light grey, n = 9) DSM strips; mean ± SD, “*” significant difference (P < 0.05).

Bladder smooth muscle is characterized by spontaneous contractile activity during the filling phase (e.g.^14^) thought to be required for facilitating adjustment of muscle bundle lengths during bladder filling^15^. Consistent with this, pre-stretched (by applying initial load of 5 mN) detrusor smooth muscle (DSM) strips from bladders of both control and BBN-treated (i.e. bladder cancer) rats displayed spontaneous contractility of quite variable amplitude and frequency (Fig. 2a).

To quantify average amplitude (*A*_SC_) and frequency (*f*_SC_ = 1/*T*_SC_, where *T*_SC_ is period) of spontaneous contractions the parts of the recordings with no change in basal tension were selected (Fig. 2a). From these parts the amplitude histogram (Fig. 2a, lower left panel) and power spectrum (Fig. 2b) were constructed. The power spectrum was used to determine the frequency of the main harmonic, which was taken as a measure of *f*_SC_, whereas the amplitude histogram was fit by the two Gaussians to obtain the best fit of the near baseline and near maximal amplitude areas. The distance between maximums of the two Gaussians was taken as *A*_SC_. With this approach quantification of *A*_SC_ and *f*_SC_ = 1/*T*_SC_ of spontaneous contractions (Fig. 2c,d) revealed that in cancerous bladders both parameters generally decrease compared to the controls (i.e. *A*_SC_ from 4.6 ± 2.9% to 2.8 ± 1.6% and *f*_SC_ from 0.047 ± 0.018 Hz to 0.027 ± 0.007 Hz in control- and cancer-DSM, respectively). Since spontaneous contractions are considered to be a factor contributing to bladder overactivity^16^, their diminution in bladder cancer may be indicative of decreased luminal pressures during filling, and detrusor underactivity.

### EFS-evoked contractions

Electric field stimulation (EFS) of bladder strips activates intramural nerve fibres causing them to release a number of neurotransmitters with contractile action on DSM. Thus, EFS largely mimics synaptically-evoked contractions that would occur naturally under nerve fibres excitation. Contraction of DSM in response to EFS (EFS-contraction) is primarily mediated by co-release of two major excitatory neurotransmitters, ATP and acetylcholine (ACh), from intramural efferent nerve endings with subsequent activation of postsynaptic purinergic P2X (P2XR) and muscarinic ACh (mAChR) receptors on DSM cells. The ratio of ATP and ACh is highly species-dependent and can change in pathological states^17,18^ with target receptors being mainly represented by ionotropic P2X1^19–21^ and metabotropic M3/M2 subtypes, respectively^22–24^. This commonly causes EFS-contraction to consist of two components linked to activation of rapidly-acting ionotropic P2X1R, and slower-acting metabotropic M3/M2 mAChR.

**Figure 3:**
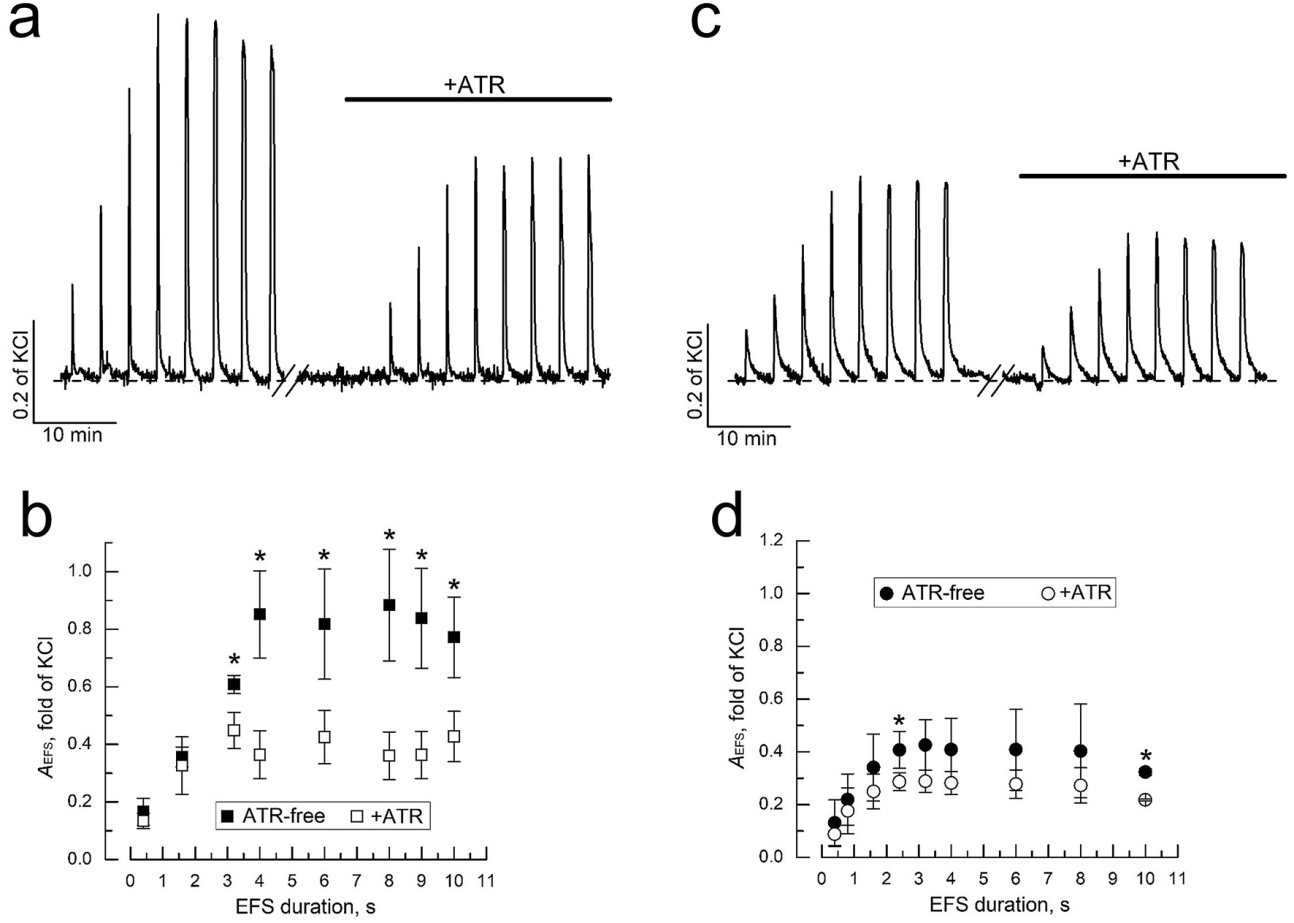
Bladder cancer down-regulates DSM contractions evoked by electric field stimulation (EFS) mostly at the expense of m-cholinergic component. (**a**) Representative original recordings of EFS-contractions of control-DSM in response to EFS of increasing duration (from left to right 0.4, 1.6, 3.2, 4, 6, 8, 9 and 10 s) before (left) and after exposure to m-cholinergic inhibitor atropine (ATR, 1 µM, right). (**b**) Quantification (mean ± SD) of the dependence of EFS-contraction amplitude (*A*_EFS_) in control DSM on EFS duration in the absence (filled symbols, n = 10) and in the presence of ATR (1 µM, open symbols, n = 10). (**c**,**d**) Same as in (**a**,**b**), respectively, but for cancer-DSM (mean ± SD, n = 10); note, the decrease of both overall *A*_EFS_ and the size of ATR-blockable component in cancer- vs. control-DSM; for (**b**,**d**) “*” significant difference (P < 0.05) between data points.

In our experiments, EFS-contraction of DSM strips from control animals could be completely inhibited by local anaesthetic and voltage-gated sodium channel inhibitor, lidocaine (2 mM), suggesting their neurogenic nature (Supplementary Fig. 1a). To test whether or not EFS-contractions change in cancer and if so what components are mainly affected we used EFS of variable duration (i.e. 0.4–10 s) in combination with m-cholinergic blocker, atropine (ATR, 1 µM). Figure 3a shows that in DSM strips from bladders of control (i.e. normal) animals (“control-DSM”) the amplitude of EFS-contractions (*A*_EFS_) progressively increased with EFS prolongation up to 4 s saturating afterwards. Application of ATR hardly changed EFS-contraction amplitude at EFS shorter than 2 s, after which ATR-blockable component increased in size, levelling at about 52% of the overall amplitude at durations longer than 4 s (Fig. 3b). This indicated that EFS-contraction mainly consists of non-cholinergic component at EFS durations below 2 s, but the contribution of m-cholinergic one increases with EFS duration maximising at 52% above 4 s.

Control experiments with P2X-receptor agonist and desensitizing agent α,β-methylene-ATP (αβ-meATP, 10 µM) have shown that the amplitude of ATR-resistant EFS-contractions by 80–90% consists of purinergic component (depending on EFS duration, Supplementary Fig. 2) with only minor contribution from other mechanism(s) most likely linked to the release of slowly acting peptide neurotransmitters (e.g.^25^).

DSM strips from animals with BBN-induced bladder cancer (“cancer-DSM”) were characterized by essentially decreased *A*_EFS_ compared to the control-DSM (Fig. 3c,d). Using ATR to dissect EFS-contraction on cholinergic and non-cholinergic components (Fig. 3c,d) revealed that in bladder cancer both ones become reduced, although to the different extents: if for m-cholinergic component the decrease constituted impressive 3.54-fold (or by 71.7%) compared to the control-DSM then for non-cholinergic one it was only 1.38-fold (or by 27.4%).

Thus, bladder cancer weakens parasympathetic stimulation of detrusor smooth muscle mainly at the expense of m-cholinergic excitatory transmission. This would cause difficulty in bladder emptying and weakening of urine stream, the symptoms typical of urologic complications in bladder cancer^4^.

### TRPV1-dependent contractility

TRPV1 is widely known as an ion channel activated by noxious heat (t ≥ 42 °C) and chemical imitator of burning sensation, capsaicin (CAP). It is mainly localized in the subset of peripheral sensory neurons involved in pain sensation^26^. However, TRPV1 expression was also found in several other neuronal and non-neuronal tissues, where its function is not always understood. There is general consensus that in the bladder TRPV1 is expressed in bladder-innervating nociceptive C-fibres responsible for the perception of pain and afferent limb of micturition reflex^27^. Whether or not it is also present on functionally-relevant levels in other bladder tissues, first of all in the urothelium, still remains the matter of controversy^27^. On the local level, TRPV1 activation in CAP-sensitive, TRPV1-expressing bladder afferents may induce DSM contraction via their “efferent” functions through the release of tachykinins, neuropeptides with contractile action on DSM^25,28^. Thus, while TRPV1 sensory function is responsible for the perception of pain and afferent limb of micturition reflex, its “efferent” activity is involved in the local control of nerve excitability and smooth muscle contractility.

**Figure 4:**
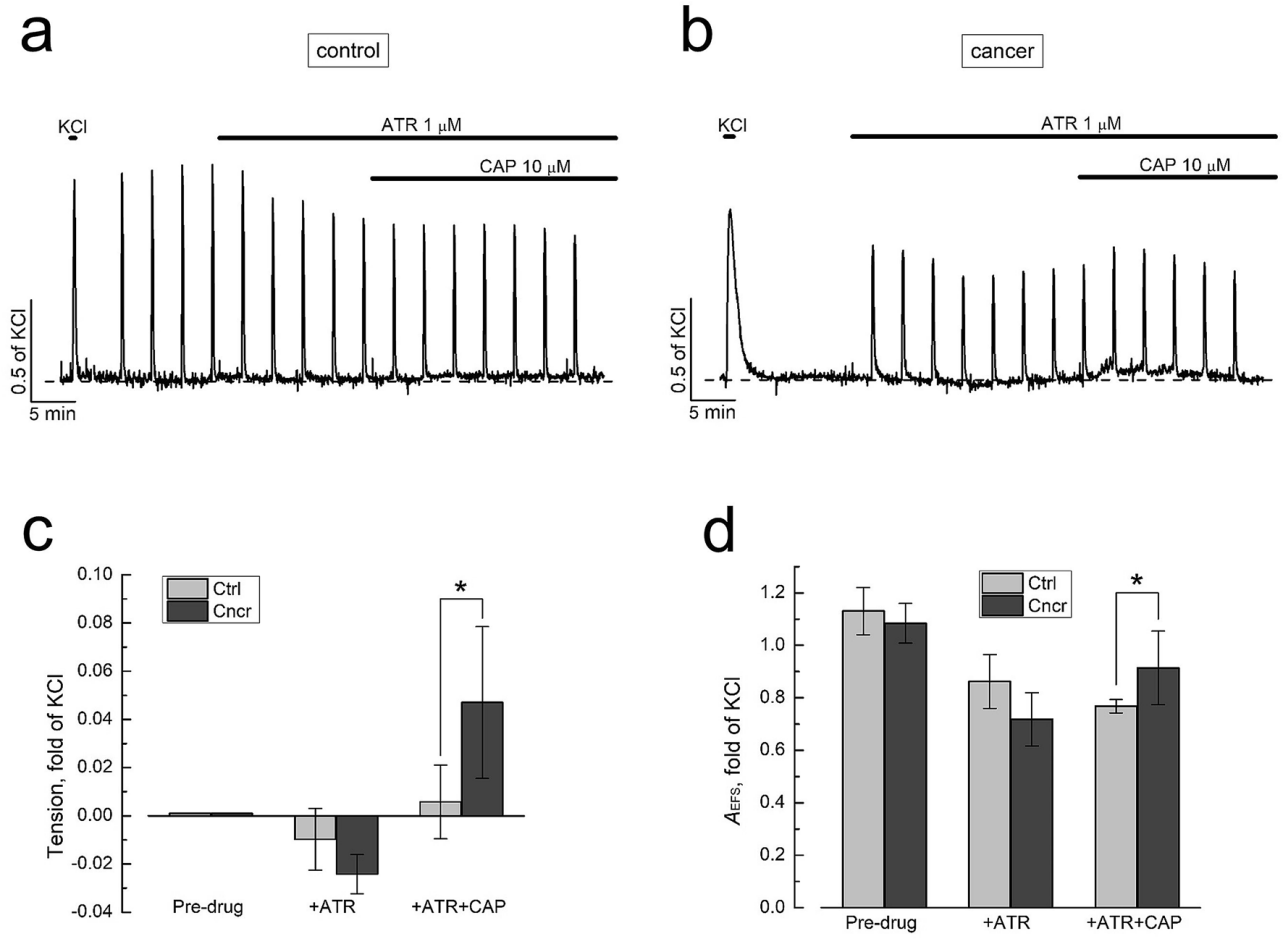
Bladder cancer up-regulates DSM contractility dependent on activation of heat and capsaicin-sensitive TRPV1-channel. (**a,b**) Representative original recordings of normal (control, **a**) and cancerous (**b**) DSM strips contractions in response to application of TRPV1 agonist capsaicin (CAP, 10 µM) on the background of atropine (ATR, 1 µM); upward spikes are EFS-contractions; note, development of stronger tension and stronger increase of EFS-contractions in cancer (**b**) vs. normal (**a**) DSM in response to CAP; in these as well as other original recordings from subsequent figures the first upward spike—contraction in response to KCl (60 mM) used for data normalization, dashed line—the level of baseline tension, thick solid lines—markers of drugs applications. (**c**,**d**) Quantification of the changes in tension (**c**) and amplitude of EFS-contractions (*A*_EFS_, **d**) at indicated interventions in normal (Ctrl, light grey bars, mean ± SD, n = 8) and cancerous (Cncr, dark grey bars, mean ± SD, n = 10) DSM strips; “*” significant difference (P < 0.05) between corresponding values; note, development of stronger tension and *A*_EFS_ enhancement in response to CAP in cancer vs. control DSM.

In our experiments, application of CAP (10 µM) on the background of ATR (1 µM, to eliminate any interference from m-cholinergic transmission) caused modest, transient enhancement of basal tension and amplitude of EFS-contraction in both control- and cancer-DSM strips (Fig. 4). Quantification of each effect revealed that in cancer-DSM CAP-induced augmentation of both basal tension and EFS-contractions was stronger than in control (Fig. 4c,d), although for EFS-contractions the difference did not rich statistical significance. In view of the fact that TRPV1 activation in isolated bladder preparations is thought to produce contractions through the release of tachykinins from CAP-sensitive TRPV1-expressing bladder afferents^25,28^, these findings suggest upregulation of TRPV1 expression, stronger TRPV1 coupling with tachykinin release machinery and/or enhancement of tachykinin-mediated DSM contractility in bladder cancer. Such alterations would favour both strengthening of TRPV1-dependent afferent limb of micturition reflex and potentiation of local TRPV1-dependent "efferent" functions of bladder afferents, consistent with the promotion of urge incontinence.

### TRPV2-dependent contractility

TRPV2-channel was initially characterized as heat sensor as well, which in contrast to TRPV1 has no sensitivity to CAP and is prominently expressed in TRPV1-negative medium- to large-diameter sensory neurons that can be activated by extreme heat (> 52 °C)^29^. Subsequent studies, however, have established that TRPV2 has little to do with temperature sensing, displaying complex pattern of expression and physiological functions^30^. Among the latter participation in osmo- or mechanosensory mechanisms in various cell types (including neurons, vascular smooth muscle, endothelial cells, immune-related cells), autonomous regulation, somatosensation, food and fluid intake, cardiovascular functions and insulin secretion^30^. TRPV2 is also implicated in carcinogenesis^31,32^.

In urinary bladder of various species the presence of TRPV2 on mRNA and protein levels was detected in nerve fibres, urothelial, suburothelial cells and smooth muscle cells, although its functional significance is bladder tissues still remains unclear^11,33^. It has been suggested that TRPV2 may act as urothelial stretch sensor and play pivotal role in the development of urothelial carcinoma^11,31^.

In our hands, application of TRPV2 agonist cannabidiol (CBD, 50 µM) caused increase of basal tension and enhancement of the spontaneous contraction amplitude with both effects being insignificantly smaller in cancer vs. control DSM (Supplementary Fig. 3).

### TRPV4-dependent contractility

TRPV4 was first described as osmosensing, volume-regulatory Ca^2+^-permeable cationic channel, mediating cell responses to changing osmotic conditions in mammals. Nowadays, however, this view has evolved to a concept of polymodal ionotropic receptor activated by multiplicity of stimuli, ranging from hypotonicity to heat and acidic pH^34^. TRPV4 is one of the most implicated channels in bladder function mostly as urothelial stretch sensor and mediator of stretch-evoked ATP release^34–36^, with possible species-specific expression and function in other bladder tissues (DSM, interstitial cells, vascular endothelium) as well^37–41^.

**Figure 5:**
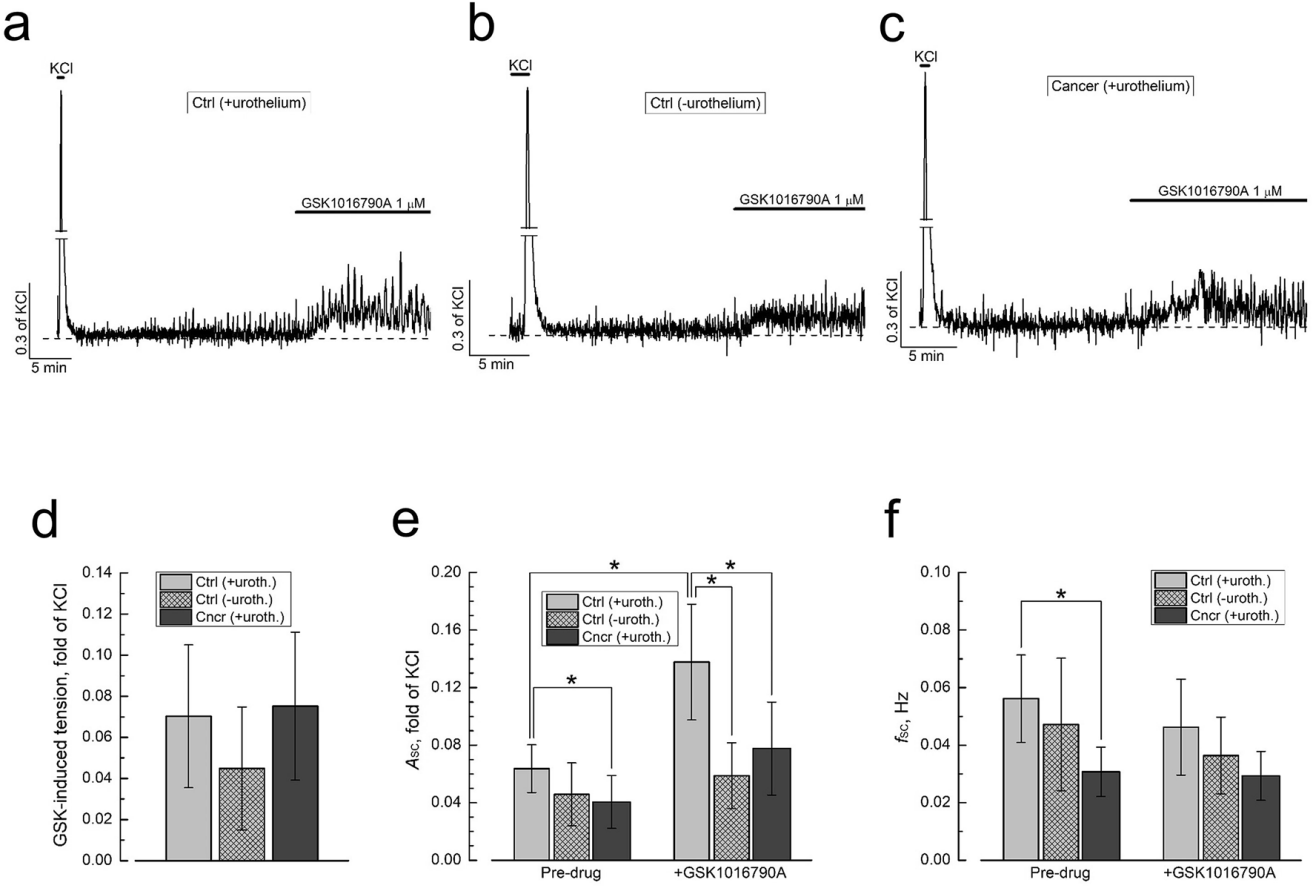
TRPV4-dependent contractility is largely preserved in mucosa-striped normal-DSM and is reduced in cancer-DSM. (**a**–**c**) Representative recordings of contractions of control DSM strips (i.e. from normal rats) with intact (+ urothelium, **a**) and removed (-urothelium, **b**) urothelium, and cancer DSM strips with intact (+ urothelium, **c**) in response to the application of TRPV4 agonist GSK1016790A (1 µM). (**d**–**f**) Quantification of the changes in GSK1016790A-induced tension (**d**), amplitude (*A*_SC_, **e**) and frequency (*f*_SC_, **f**) of spontaneous contractions in normal (Ctrl) urothelium-preserved (+ uroth., light grey bars, mean ± SD, n = 10) and urothelium-devoid (-uroth., hatched light grey bars, mean ± SD, n = 10) DSM strips and in cancer-DSM strips (Cncr) with intact urothelium (+ uroth., dark grey bars, mean ± SD, n = 9); “*” significant difference (P < 0.05) between corresponding values.

We studied TRPV4-dependent DSM contractility using TRPV4 agonist GSK1016790A^38^ with Fig. 5a–c presenting examples of original recordings and Fig. 5d–f quantification of the results. In control DSM strips with intact mucosa (Fig. 5a) GSK1016790A (1 µM) induced two effects: (1) transient enhancement of basal tension (Fig. 5d) and (2) increase of the amplitude of spontaneous contractions (*A*_SC_, Fig. 5e) without essentially affecting frequency of spontaneous contractions (*f*_SC_, Fig. 5f). Removal of urothelium (Fig. 5b) decreased the amplitude of tension enhancement in response to GSK1016790A by about 36% (i.e. from 0.07 ± 0.034 to 0.045 ± 0.03, Fig. 5d) and effectively reduced *A*_SC_ enhancement by GSK1016790A (i.e. from about 2.2-fold in the presence of urothelium to only about 1.3-fold in its absence, Fig. 5e), but left *f*_SC_ virtually unaffected by drug (Fig. 5f). This indicates that contractile response to TRPV4 activation in rat bladder is by about 70% mediated by TRPV4 localized in DSM and probably interstitial cells, as was postulated before for mouse bladder^38^, and by about 30% by urothelium-dependent effects. Besides, activation of urothelial TRPV4 appeared to contribute to spontaneous contractility.

Exposure of cancer-DSM strips to GSK1016790A (Fig. 5c) produced enhancement of basal tension of nearly the same magnitude as in control ones (Fig. 5d), but failed to augment *A*_SC_ as potently as in control-DSM (Fig. 5e) and completely lost the ability to influence *f*_SC_ (Fig. 5f). The magnitude of tension enhancement is determined by the global increase of intracellular Ca^2+^ concentration ([Ca^2+^]_i_) in DSM cells which includes Ca^2+^ entry and Ca^2+^ release components, whereas parameters of spontaneous contractions correlate with electrical activity which in turn is function of membrane potential (V_m_), increasing with depolarization^15^. Thus, our findings indicate that bladder cancer does not affect global TRPV4-dependent [Ca^2+^]_i_ increase in DSM cells, but essentially reduces V_m_ depolarization associated with TRPV4 activation.

### Involvement of Ca^2+^-activated K^+^-channels

K^+^-channels play important roles in regulation of DSM cells excitability and contractility^9^. Of a number of K^+^-channel types present in DSM cells the so called BK one (also known as Slo, Slo1, maxi K, K_Ca_1.1), belonging to the Ca^2+^-activated K^+^-channel family^42^ displays the highest level of expression and arguably is the most important physiologically relevant K^+^-channel in DSM of all species^9^.

**Figure 6:**
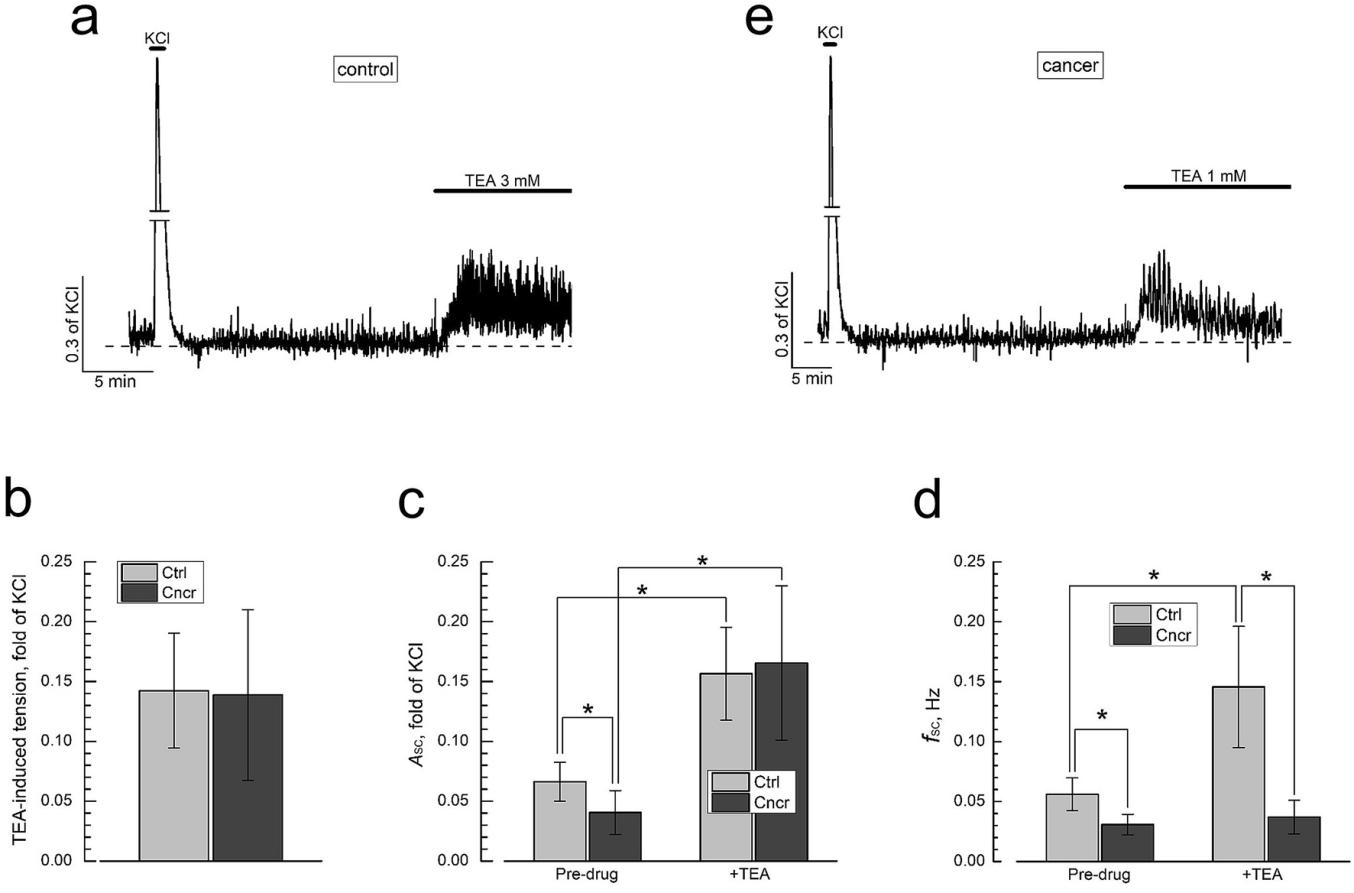
Bladder cancer eliminates BK channel-dependent modulation of spontaneous contractions frequency. (**a**,**b**) Representative recordings of contractions of DSM strips from normal (control, **a**) and cancerous (**b**) bladder in response to application of BK-channel blocker TEA (3 mM); note, strong increase of spontaneous contractions frequency in response to TEA in control DSM (**a**) and lack thereof in cancer one (**b**). (**c**–**e**) Quantification of the changes in TEA-induced tension (**c**), amplitude (*A*_SC_, **d**) and frequency (*f*_SC_, **e**) of spontaneous contractions in control (Ctrl, light grey bars, mean ± SD, n = 10) and cancerous (Cncr, dark grey bars, mean ± SD, n = 8) DSM strips; “*” significant difference (P < 0.05) between corresponding values.

Figure 6a–d shows that exposure of the control-DSM strip to the non-specific BK-channel inhibitor TEA (3 mM)^42^ transiently enhanced basal tension, and strongly increased both *A*_SC_ and *f*_SC_, indicating that BK blockade and associated DSM depolarization brings about global [Ca^2+^]_i_ rise as well as promotes burst-type electrical activity within the strip, underlying spontaneous contractility, through the increase of the frequency and duration of burst firing^15^. In view of the fact that TEA is non-specific BK-channel inhibitor, contribution of other types of potassium channels expressed in DSM^9^ to these effects cannot be excluded.

In cancer-DSM (Fig. 6e), TEA appeared basically as effective as in control ones in enhancing basal tension and augmentation of *A*_SC_ (Fig. 6b,c), but completely failed to increase *f*_SC_ (Fig. 6d). This suggests that bladder cancer has little effect on global intracellular Ca^2+^ signalling in DSM cells, but impairs V_m_-dependent generation of periodic electrical and mechanical activity within DSM and interstitial cell syncytium^15^. This conclusion corresponds with the observations of TRPV4-dependent contractility.

## Discussion

Our data show that bladder cancer induces multifactorial changes in DSM contractility, most of which are consistent with the symptomatic of bladder dysfunction in bladder cancer patients^4^. The most obvious and straightforward alteration in cancer-DSM contractility concerned spontaneous contractions with both *A*_SC_ and *f*_SC_ being essentially reduced in cancer vs. normal DSM. The cellular basis of spontaneous contractions in DSM are still uncertain, but available data mostly point to their myogenic origin with activity of multiple ion channels and intracellular signalling pathways taking part in shaping their amplitude and temporal characteristics^15,16,43^. Given the complexity of the mechanism underlying spontaneous contractions, further studies are needed to determine the exact reason(s) of their reduction in bladder cancer. Normally, spontaneous activity of DSM contributes to the luminal pressure and works against the bladder compliance (i.e. increasing volume with little change in bladder pressure) in the filling phase. Its decrease in bladder cancer is indicative of decreased pressure and increased compliance, potentially leading to overflow incontinence due to filling beyond the limits that bladder can hold.

Another characteristic feature of cancer-DSM was reduced amplitude of EFS-contractions compared to normal DSM, occurring mostly at the expense of m-cholinergic component. EFS-contractions arise from electrical excitation of intramural nerve endings releasing a bunch of neurotransmitters with contractile action on DSM cells. In this context EFS provides a good approximation of natural synaptically-evoked contractions. The key neurotransmitter released from bladder efferents is ACh which is responsible for parasympathetic DSM stimulation via DSM-localized mAChR and co-released with it ATP, acting on DSM cells' purinergic P2X1R^17,18^.

One can suggest several reasons for the decreased EFS-contractions in bladder cancer: (1) the reduction in the density of cholinergic parasympathetic nerve endings, (2) impaired neurotransmitter (i.e. ACh and ATP) release and/or clearance, and (3) aberrant mAChR- and P2X-mediated signalling in DSM due to decreased expression of respective receptors or downregulation of signalling pathway(s) linked to their activation. At present it is difficult to select among these possibilities, however, irrespective of the exact reason(s), the decrease of EFS-contractions is indicative of motor deficit with concomitant difficulty in bladder emptying and weakened urine stream, the symptoms typical of urologic complications in bladder cancer^4^.

Heat and capsaicin-sensitive TRPV1 is currently viewed as mostly neuronal channel within the bladder, predominantly expressed in bladder-innervating nociceptive C-fibres responsible for the perception of pain and afferent limb of micturition reflex^27^. Thus, slight potentiation of TRPV1-dependent contractility of isolated DSM strips from cancerous bladder would be indicative of enhanced “efferent” function of TRPV1-expressing bladder afferents due to release of pro-contractile tachykinins in response to TRPV1 activation^25^. Such enhancement may result either from up-regulated TRPV1 expression in cancerous bladder afferents promoting tachykinin release or sensitization of tachykinin-dependent pathway in DSM contractility. In the event of bladder cancer-promoted neuronal TRPV1 expression one would also expect strengthening of TRPV1-dependent afferent limb of micturition reflex. Altogether, such alterations would favour decreased micturition threshold, increased perception of pain and increased local DSM contractility in response to irritants in bladder cancer, which would be generally consistent with the promotion of urge incontinence.

Although TRPV1 expression on functionally-relevant levels in urothelium still remains the matter of controversy^26^, in human urothelial cancer specimens TRPV1 mRNA and protein expression is, in fact, progressively decreasing from low grade to high-grade muscle-invasive urothelial carcinoma as compared to normal urothelium^44,45^. Thus, TRPV1 may play dual role in bladder cancer: on the one hand, the decrease of its urothelial content would promote oncogenic apoptosis resistance^46^ and, on the other hand, its enhanced function as a receptor of various sensory modalities in bladder afferents would potentiate DSM contractility.

We did not find any substantial differences in TRPV2-dependent contractility of normal vs. cancerous DSM with TRPV2 agonist, CBD, being nearly equally effective in increasing basal tension and enhancing *A*_SC_ in both types of DSM. Although TRPV2 expression was reported in several bladder tissues^11^, aside from the involvement in the development of urothelial carcinoma^11,31^, its significance in urinary bladder remains undefined. Contractile action of CBD suggests that TRPV2 may be also an important determinant of DSM contractility, although its role in the symptomatic of bladder dysfunction associated with cancer is likely to be small if any.

Of all the TRPs, the TRPV4 seems to be the most implicated in bladder function as sensor of stretch and regulator of bladder filling^34^. The presence of TRPV4 was detected in rodents' (mice, rats, guinea-pigs) urothelium, DSM, vascular endothelium and interstitial cells^37–41^ as well as in human urothelium^47^. *Trpv4-*knockout mice demonstrate aberrant voiding pattern and stretch-evoked ATP release from urothelium^37^. Intravesical instillation of TRPV4 specific agonist, GSK1016790A, was shown to increase firing of mechanosensitive, CAP-insensitive bladder afferents in rats^39^ and to promote bladder overactivity in mice and rats^38,48^.

Broad TRPV4 expression in bladder tissues implies diversity and species-specificity of its engagement in bladder contractility. Indeed, by supporting stretch-evoked ATP release from urothelium it may communicate distention signal to underlying P2XR-expressing bladder afferents, facilitating the micturition reflex^35,39^, whereas by being present in DSM cells it may contract DSM directly^38^ and/or regulate stretch-dependent DSM excitability and spontaneous activity through coupling with Ca^2+^-dependent K^+^-channels^40,41^.

In our experiments on normal DSM, GSK1016790A caused transient enhancement of basal tension and increased *A*_SC_. The first effect was only about 30% smaller and second one was virtually unchanged in mucosa-denuded DSM strips. As experimentation on isolated DSM strips excludes neuronal-mediated effects, this suggests that: (1) rat DSM cells express functional TRPV4 whose activation can cause global [Ca^2+^]_i_ increase, (2) overall GSK1016790A-evoked tension includes two components, the one linked to TRPV4-dependent release of ATP from urothelium with its subsequent action on DSM P2X1 receptors, and the one associated with direct activation DSM-localized TRPV4, (3) activation of DSM-localised (and probably interstitial cell-localised) TRPV4 causes depolarization promoting burst lengthening within the periodic burst-type electrical activity underlying spontaneous contractility^15^.

BBN-induced bladder cancer in rat did not affect the magnitude of GSK1016790A-evoked tension, but reduced the extent of *A*_SC_ augmentation by GSK1016790A. This suggests that cancer has little or no influence on TRPV4-dependent global [Ca^2+^]_i_ elevation in DSM cells, but impairs the mechanisms responsible for V_m_ depolarization and generation of electrical bursting activity.

It should be noted, though, that TRPV4 expression in various cell types in the bladder and its coupling to the V_m_ changes, electrical activity and DSM contractility seem to be species specific. Moreover, if there is general consensus as to urothelial expression and function of TRPV4 then its presence and role in DSM remains controversial. This especially concerns mouse bladder for which reports on TRPV4 mRNA and protein expression in DSM^38^ are interlaced with the ones showing lack thereof^37,41^. The results of functional studies using GSK1016790A are also divergent: GSK1016790A was reported to induce contraction and *A*_SC_ increased^38^ as well as relaxation and *A*_SC_ decrease^41^ of urothelium-devoid mouse DSM strips.

In guinea-pig bladder TRPV4 was detected in DSM and muscularis mucosae (smooth muscle layer between urothelium and DSM) with GSK1016790A producing sustained contraction and not enhancement, but cessation of spontaneous activity in both tissue types^40^. In the events when TRPV4 activation was accompanied by spontaneous contractions inhibition, TRPV4 coupling to SK^41^ and BK^40^ Ca^2+^-dependent K^+^-channels with ensuing V_m_ hyperpolarization and decrease of electrical excitability was implicated. Such coupling was postulated to play role in preventing bladder overactivity during filling and to act as a self-limiting mechanism for bladder contractility during its storage phase.

Pharmacological blockade of BK channels with TEA in normal rat DSM enhanced basal tension, and strongly increased both *A*_SC_ and *f*_SC_ (see Fig. 6), consistent with DSM cells V_m_ depolarization, activation of voltage-gated Ca^2+^ influx and enhancement of burst-like electrical activity. We did not find any interdependence or mutual influence of GSK1016790A and TEA responses in the experiments with combined drugs applications (data not shown), suggesting no interaction of TRPV4 and BK channels in rat bladder in contrast to the guinea-pig one^40^. Compromised ability of TEA to influence *A*_SC_ and *f*_SC_ in cancer-DSM (see Fig. 6) indicates that cancer primarily affects the mechanisms of periodic burst-type electrical activity underlying spontaneous contractility.

## Methods

### Rat model of bladder cancer

All animal protocols complied with EU Directive 2010/63/EU for animal experiments^49^. The protocol was approved by Bogomoletz Institute of Physiology Bioethics Committee (Permission No 1/17 from 26.06.2017). Bladder cancer induction in rats generally followed the procedure described by Vasconcelos-Nóbrega et al.^12^. Wistar male rats weighing 200–250 g were treated with urothelial carcinogen, BBN (Sigma-Aldridge), through oral rout of administration with drinking water (0.05% w/v). The treatment lasted for 4 months (16 weeks) followed by 2 weeks rest and 2 weeks experimental period. Animals receiving BBN with drinking water constituted experimental bladder cancer group with age-mates receiving regular drinking water serving as controls. Three different groups of 6, 10 and 4 experimental animals were used in the experiments described in this study.

### Bladder strips preparation and contraction measurements

Animals were anesthetized by brief carbon dioxide exposure and sacrificed by decapitation. Whole urinary bladder was removed and placed in the warmed (37 °C), oxygenated (95% O_2_ and 5% CO_2_) Krebs solution (in mM): 120.4 NaCl, 5.9 KCl, 1.2 MgCl_2_, 1.2 NaH_2_PO_4_, 1.8 CaCl_2_, 15.5 NaHCO_3_, 11.5 glucose (pH 7.4). Under stereo microscopic control the bladder was cut ventrally from base to dome and separated onto two parts in approximate 1/3 to 2/3 proportion. The smaller part was spared for histopathological evaluation, and the larger part was mechanically cleaned from mucosa (when necessary) using microsurgery scissors and cut onto longitudinal strips (diameter ~ 2 mm, length ~ 7–10 mm) for tensiometric contraction measurements. Only experiments aimed at assessing how the presence of mucosa influences the responsiveness to TRPV4-channel activator GSK1016790A in control rats were conducted on both mucosa-intact and mucosa-free DSM strips. All other contractility studies from control and BBN-treated animals were performed on DSM strips with intact mucosa.

For contraction measurements the strip was positioned in organ bath continuously superfused with preheated experimental solutions (37 °C) with one end fixed still and another end attached to the capacitative force sensor^50^. Standard electric field stimulation (EFS) protocol consisted of 2-s-long train of pulses (pulse duration 0.5 ms, amplitude 100 V, frequency 10 Hz) applied every 3 min via two Ag/AgCl electrodes flanking the strip. Contractile activity recording was made using pCLAMP software and DigiData 1200 to the computer and in parallel on pen recorder.

All chemicals used in the study were from Sigma-Aldrich and were added to the experimental Krebs solution from respective stock solutions. Atropine (ATR) was dissolved in water as 10 mM stock, capsaicin (CAP, TRPV1 agonist), cannabidiol (CBD, TRPV2 agonist) were dissolved in ethanol as 10 mM stocks, GSK1016790A (TRPV4 agonist) was dissolved in DMSO as 10 mM stock. Control experiments indicated that solvents in the concentration up to 0.1% did not produce any effect on DSM strips contractility.

### Histopathological examination

Bladder tissues were fixated in the 4% solution of formaldehyde, dehydrated in the increasing ethanol concentrations, cleared with benzene and embedded in paraffin boxes. Paraffin embedded specimens were sliced (5 μm thick) on a microtome and attached to slides using albumin. Slices were deparaffinized with benzene, rehydrated with decreasing ethanol concentrations, stained with H&E, dehydrated with increasing ethanol concentration, cleared with benzene and covered with glass coverslips using Canada balsam (Sigma-Aldrich).

### Data analysis and statistics

Total of 18 bladder cancer rats were used in this study (2 rats out of 20 receiving BBN in drinking water did not develop discernible bladder cancer lesions). Each functional experiment was performed on 6–10 DSM strips (n) from at least 3 animals (N). Contractile responses of the strips even from the same bladder essentially depended on whether or not the part of the bladder the strip was excised from contained discernible under low magnification stereomicroscope cancer lesion(s) and the size of those lesion(s) (see Fig. 1c). To enable averaging of contraction amplitudes generated by the strips within the same group of animals and their comparison between different groups they were normalized to the amplitude of KCl (60 mM) contraction of the same strip. Control calculations in which amplitude of KCl contraction was normalized to the strip weight have shown that it does not show statistically significant difference between control bladder cancer groups (Supplementary Fig. 1b). The parameters of the contractile responses to the same intervention in control and bladder cancer-derived DSM strips were measured, averaged and expressed as mean ± SD with the number of studied strips indicated by “n”. Statistical comparison of the data between control and bladder cancer groups was made by unpaired t-test with P < 0.05 was considered significant. In view of essential variability of lesions' quantity and sizes within DSM strips from cancerous bladders as well as variability in their contractile responses, the data of the same type obtained from all cancer bladders were pooled for statistical purposes. No correlation to the number and size of cancer lesions was made.

## Supplementary information

Supplementary Information

## Data Availability

The datasets from the current study are available from the corresponding author on reasonable request.
