# Supplementary material for: Alterations in detrusor contractility in rat model of bladder cancer
Source: Sci Rep. 2020 Nov 12;10:19651. doi: 10.1038/s41598-020-76653-7 (PMC7665011; doi:10.1038/s41598-020-76653-7)
Supplement: Supplementary file 1 — Supplementary Information [file 41598_2020_76653_MOESM1_ESM.pdf]

SUPPLEMENTARY INFORMATION

Alterations in detrusor contractility in rat model of bladder cancer

Igor B. Philyppov[1], Ganna V. Sotkis[1], Aurelien Rock[2], Morad Roudbaraki[2], Jean-Louis Bonnal[2], Brigitte Mauroy[2], Natalia Prevarskaya[2], Yaroslav M. Shuba[1]

[1]Bogomoletz Institute of Physiology of the National Academy of Sciences of Ukraine, Kyiv, Ukraine;

[2]Laboratory of Cell Physiology, Inserm U1003, University of Lille, Villeneuve d'Ascq, France

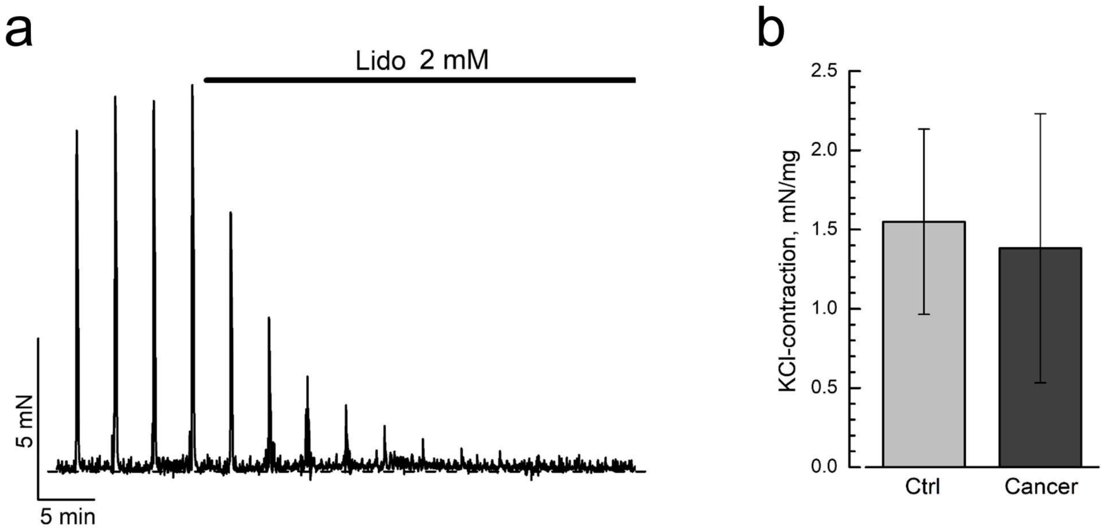

Supplementary Figure 1. Validation of neurogenic nature of EFS-contractions and KCl normalization procedure. **a:** Representative recording of EFS-contractions of control-DSM in response to 2 s long EFS showing complete suppression of the contractions by local anaesthetic and voltage-gated sodium channel inhibitor, lidocaine (Lido, 2 mM, time of application marked by thick, solid, horizontal line). **b:** Bar graph showing no statistically significant differences between KCl-contraction amplitudes normalized to the DSM strip weight from control (light grey bar) and BBN-treated (dark grey bar, bladder cancer group) rats; mean±SD, n=30 for both groups.

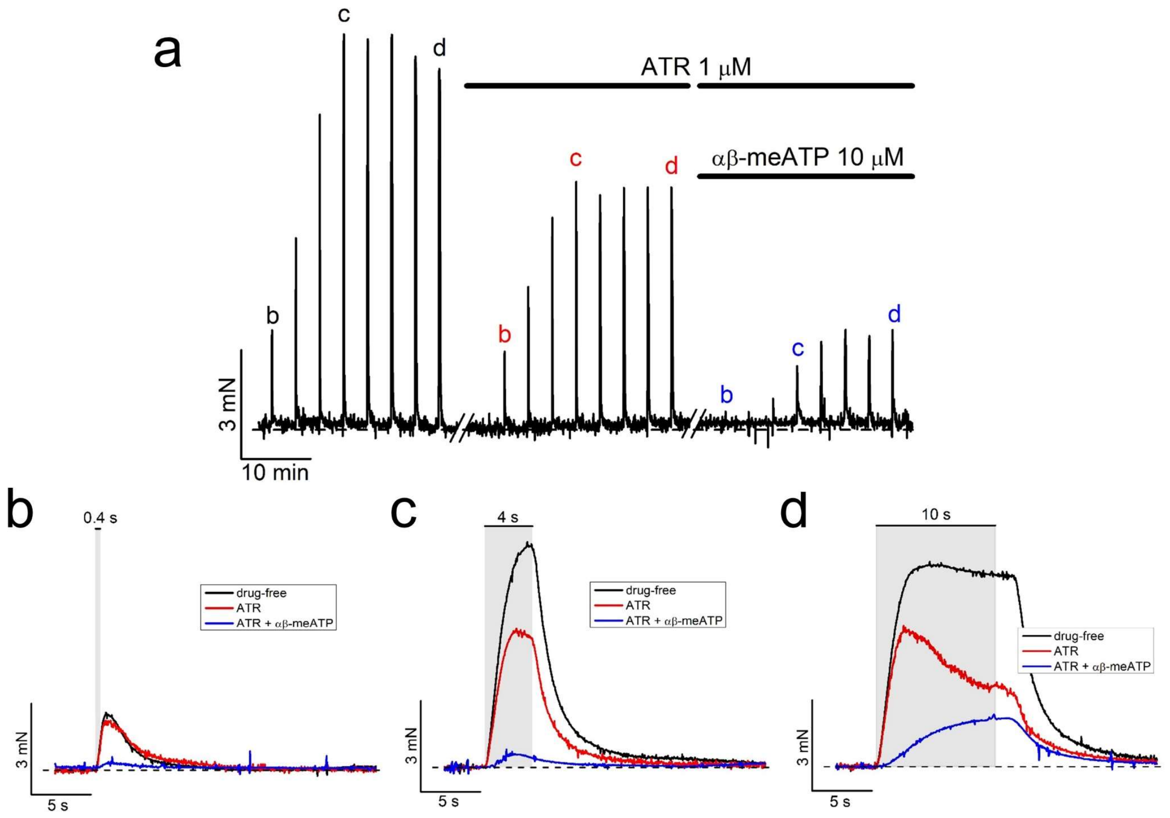

Supplementary Figure 2. Dissection of EFS-contractions onto m-cholinergic, purinergic and residual components. **a:** Representative original recordings of EFS-contractions of control-DSM in response to EFS of increasing duration (from left to right 0.4, 1.6, 3.2, 4, 6, 8, 9 and 10 s) under drug-free conditions (left), in the presence of m-cholinergic inhibitor atropine (ATR, 1 μM, middle) and in the presence of ATR plus P2X-receptor agonist and desensitizing agent α,β-methylene-ATP (αβ-meATP, 10 μM, right); drug applications are marked by thick solid horizontal lines. **b-d:** expanded views on superimposed EFS-contractions evoked by 0.4 s (**b**), 4 s (**c**) and 10 s (**d**) EFS (marked by solid line on top and shaded area) under drug-free (black line), ATR (red line) and ATR+αβ-meATP (blue line) conditions; recordings of panels **b-d** are marked by the same color-coded letters in panel **a**; note the presence of residual, slowly developing, low amplitude component of EFS contractions after suppression of m-cholinergic and purinergic components.

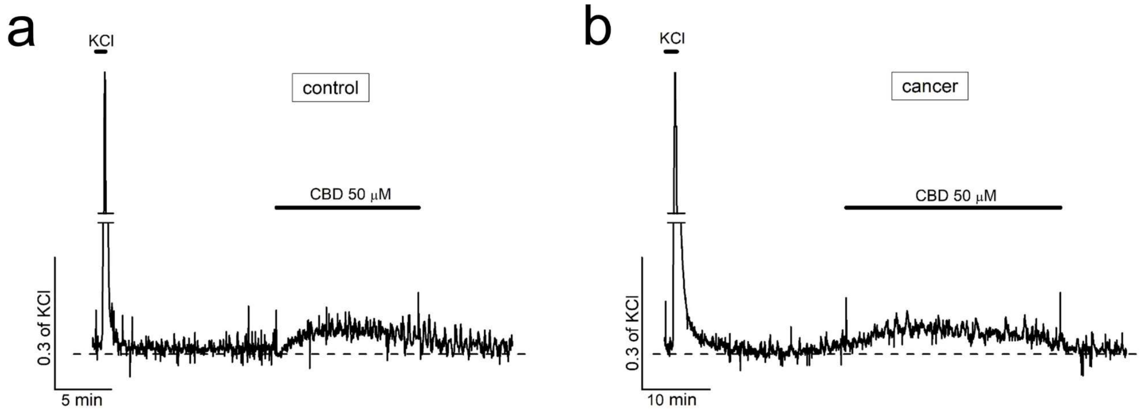

Supplementary Figure 3. Bladder cancer does not significantly influence TRPV2-dependent DSM contractility. **a, b:** Representative original recordings of contractions in response to application of TRPV2 agonist cannabidiol (CBD, 50 μM) in normal (i.e. control, **a**) and cancerous (**b**) DSM strips; note, almost no difference in the tension increase and change in the amplitude or frequency of spontaneous contractions in response to CBD between cancerous (**b**) and normal (**a**) DSM.
